# Improvement of Oxazolone-Induced Ulcerative Colitis in Rats Using Andrographolide

**DOI:** 10.3390/molecules25010076

**Published:** 2019-12-24

**Authors:** Liuhong Zhang, Ning Cao, Yuwen Wang, Youxu Wang, Chao Wu, Xuemei Cheng, Changhong Wang

**Affiliations:** Institute of Chinese Materia Medica, Shanghai University of Traditional Chinese Medicine, The MOE Key Laboratory for Standardization of Chinese Medicines, Shanghai R&D Centre for Standardization of Chinese Medicines, 1200 Cailun Road, Shanghai 201203, China; mine90@163.com (L.Z.); 18616024782@163.com (N.C.); wyxzd1314@163.com (Y.W.); vera105370@163.com (C.W.); chengxuemei1963@163.com (X.C.)

**Keywords:** andrographolide, oxazolone, ulcerative colitis, IL-4, IL-13, IL-4R/STAT6

## Abstract

Ulcerative colitis (UC) is usually accompanied with symptoms of abdominal pain, diarrhea, and bloody stool, which impair the quality of life of patients. Previous studies have shown that *Andrographis paniculata* extracts, which have andrographolide (AND) as their main compound, can relieve UC symptoms in patients. The aim of the study was to investigate the alleviating effect of AND on UC using the oxazolone (OXZ)-induced UC rat model. A total of 66 healthy male Sprague Dawley rats were used to evaluate the efficacy and mechanism of AND on UC (n = 11 per group) and grouped into control, model, SASP (sulfasalazine, positive control group, 500 mg/kg), AND-L (40 mg/kg), AND-M (80 mg/kg), and AND-H (120 mg/kg). The colonic disease activity index (DAI), colon length, spleen coefficient, pathological damage, and inflammation-related cytokine and protein expression levels were used as indices for evaluation. Results showed that the AND groups had reduced DAI and mortality, and significantly improved colon length and spleen coefficient compared with the model group. Furthermore, OXZ-induced histological injury was relieved significantly after AND treatment due to an improved crypt structure and reduced infiltration of inflammatory cells. Moreover, AND inhibited myeloperoxidase (MPO) activity and the secretion of interleukin-4 (IL-4), IL-13, and tumor necrosis factor α (TNF-α). The results of the anti-inflammatory mechanism revealed that AND blocked the signal transduction by reducing IL-4/IL-13 specific binding to IL-4 receptor (IL-4R) and inhibiting the phosphorylation of the signal transducer and activator of transcription 6 (p-STAT6). In conclusion, aside from natural plants, AND may be a candidate ingredient for UC therapy.

## 1. Introduction

Ulcerative colitis (UC) is a highly progressive inflammatory disease accompanied by symptoms, such as weight loss, abdominal pain, and bloody diarrhea [1]. Currently, adrenocorticotropic hormones and anti-infective and immunosuppressive agents are the common clinical treatments for UC. Nevertheless, some patients, especially those with refractory UC, show no sign of improvement after treatment with the current drugs, whereas others exhibit side effects or complications [2]. Accordingly, attention has been given to dietary supplements and natural products as alternative therapies for patients who do not respond to standard medications [3,4,5].

Andrographolide (AND), a major active constituent of the medicinal plant *Andrographis paniculata*, has been used in China for a long time because of its heat-clearing, anti-inflammatory, detumescence, and acesodyne effects [6]. AND-containing mixtures are mainly used to treat upper respiratory tract infection and bacterial dysentery in a clinical setting [6]. In recent years, the use of AND in the treatment of inflammatory bowel disease has attracted attention. The extract from *A. paniculata* (HMPL-004), in which AND is the main compound, can treat patients with mild to moderate UC by alleviating clinical symptoms and promoting mucosal healing [7,8]. Also, HMPL-004 can prevent the development of T-cell-dependent murine colitis [9]. A series of experiments has been designed to verify the improvement of UC due to AND. Based on the clinical samples, the percentages of Th17 cells in CD4^+^ cells increase in patients with UC compared with those in healthy individuals. Hence, the aim of the experiments was to study the Th17 immune responses involved in the pathogenesis of UC [10]. AND can inhibit the activity of the interleukin (IL)-23/IL-17 axis and decrease the expression levels of tumor necrosis factor α (TNF-α), IL-1β, IL-6, and IL-17A in the serum to suppress an inflammatory response [10]. Moreover, the peripheral blood mononuclear cells isolated from patients with UC and healthy people have been used to study the effect of AND on Th1/Th2/Th17 responses, which suggests that AND may potentially treat IL-23-mediated diseases [11]. In addition, the AND derivative CX-10 ameliorates UC induced by dextran sulfate sodium through inhibiting the activation of the nuclear factor kappa-B (NF-κB) and the mitogen-activated protein kinase (MAPK) pathways [12].

Several researchers believe that an ideal model for studying the pathogenesis of UC should have excessive production of Th2 cytokines [13]. The oxazolone (OXZ)-induced colitis model is based on a Th2-driven immune response and manifests mucosal inflammation limited to colonic mucosa and submucosa, especially in the distal colon [13,14]. Moreover, the disease features of OXZ-induced colitis model in animals are similar to those of human UC. These features include epithelial cell loss, depletion of goblet cells, inflammatory cell infiltration, edema formation, hemorrhage, and vascular dilation [15]. Also, in this animal model, the upregulation of key immunoregulatory cytokines IL-4 and IL-13 is attributed to lesions due to reduced transepithelial resistance [16,17]. Thus, IL-4 or IL-13 neutralization can ameliorate this disease [14,18,19]. The possible mechanism involved in the inflammatory response of IL-4 and IL-13 is the regulation of gene transcription by activating the signal transducer and activator of transcription 6 (STAT6) [20,21]. 

In this study, the therapeutic effect of AND on an OXZ-induced UC model was investigated. The main evaluation indices used were the colonic disease activity index (DAI), colon length, spleen coefficient, myeloperoxidase (MPO) activity, pathological damage assessment, and the expression levels of IL-4, IL-13, and TNF-α. Furthermore, Western blot analysis was used to study the preliminary mechanism of AND reversing the OXZ-induced UC by detecting the expression of the activation of STAT6.

## 2. Results

### 2.1. Effect of AND on DAI Score and Inflammatory Markers

**Figure 1:**
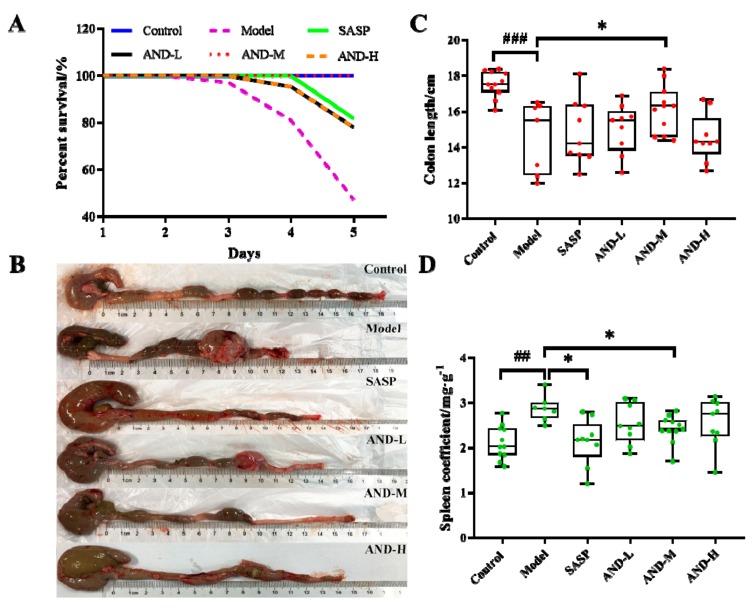
Effect of AND on oxazolone (OXZ)-induced colitis. (**A**) Survival rate of experimental rats. (**B**) Colon morphology at the end of experiment. (**C**) Colon length of experimental rats at the end of experiment. (**D**) Spleen coefficient of experimental rats (n = 7–11, control and AND-M groups: n = 11; model group: n = 7; AND-L, AND-H, and SASP group: n = 9). ^###^ *p* < 0.001 and ^##^ *p* < 0.01 relative to the control group; * *p* < 0.05 relative to the model group. (AND: andrographolide; SASP: sulfasalazine; AND-L: 40 mg/kg AND; AND-M: 80 mg/kg AND; AND-H: 120 mg/kg AND.) The data distribution of colon length and spleen coefficient did not meet the requirements of a normal distribution.

Compared with the control group, the model group had a significantly higher DAI score, which indicated the establishment of experimental colitis. The administration of sulfasalazine (SASP) and 80 and 120 mg/kg AND reduced the colon injury, as shown in Table 1. However, the DAI scores of the AND-L and the model groups were not different, indicating that the administration of 40 mg/kg AND did not affect the DAI score. In the model group, animal deaths started on the third day after OXZ administration, and the animal mortality was 41.7% by the last day. As expected, 80 mg/kg AND was found to prolong the survival time of experimental animals on the basis of the statistical analysis of the mortality of each group (Figure 1A). Interestingly, the mortality of the AND-H (120 mg/kg) group was higher than the AND-M (80 mg/kg) and the AND-L (40 mg/kg) groups, and further research was needed to determine the reason. Splenomegaly and colon shortening are reported as the characteristic markers of UC [22]. The apparent shortening of the colon was observed in the model group, and the oral administration of 80 mg/kg AND significantly relieved this symptom (Figure 1B,C). In addition, splenomegaly was significantly increased in the model group compared with the control group, which indicated the abnormality of the immune function (Figure 1D). The oral administration of SASP and 80 mg/kg AND significantly reduced the degree of splenomegaly relative to the model group. However, no significant improvement was observed in colon shortening and splenomegaly in the AND-L and the AND-H groups compared with the model group.

### 2.2. Hematoxylin and Eosin (H&E) Staining and Immunohistochemical Analysis

**Figure 2:**
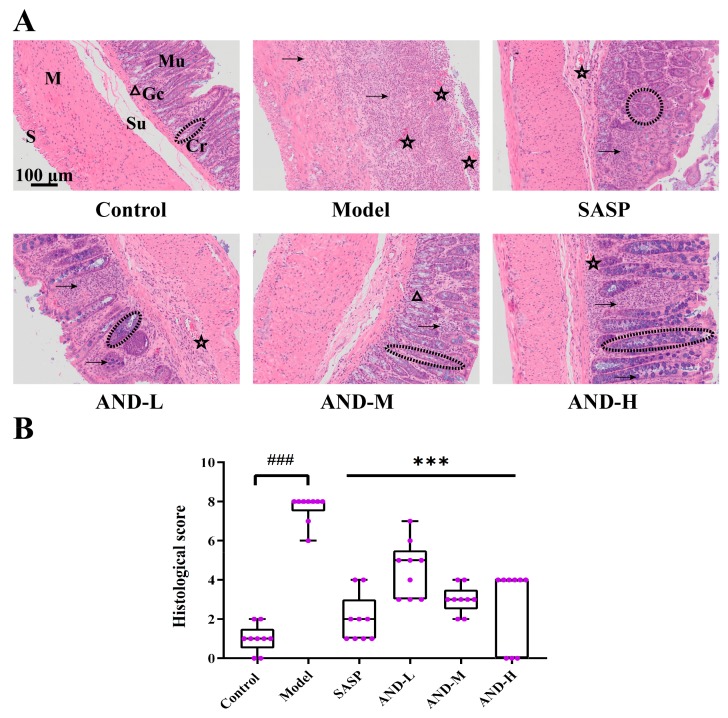
Histologic examination of colonic tissues. (**A**) Histologic magnification (×100 μm). Mu: Mucosa; Su: Submucosa; M: Muscle; S: Serosa; Cr: Crypt; Gc: Goblet cell. → Inflammation, △ Goblet cell, ☆ Edema. The black dotted lines highlight the crypt. (**B**) Pathological scores. Data are reported as means ± SD, n = 9. ^###^ *p* < 0.001 vs. the control group; *** *p* < 0.001 vs. the model group. (AND: Andrographolide; SASP: Sulfasalazine; AND-L: 40 mg/kg AND; AND-M: 80 mg/kg AND; AND-H: 120 mg/kg AND) The data distribution of pathological score did not meet the requirements of a normal distribution.

The histologic examination of the colonic sections (×100 µm) is shown in Figure 2A, and the pathological scores are illustrated in Figure 2B. The transverse section of the colon was composed of mucosa, submucosa, muscle, and serosa layers from inside to outside. The colon structure of the control group was clear, and normal colonic glands were abundantly filled with mucin-secreting goblet cells. Furthermore, only a few inflammatory cells in the mucosa layer were present, but no inflammatory infiltration was observed in the other layers. As shown in Figure 2A, the basic structure of the colon of the model group was absolutely destroyed and infiltrated with inflammatory cells from the mucosa layer to the muscle layer. After the administration of SASP and AND, the OXZ-induced histological injury was relieved significantly. The transverse sections of the colon from the AND-L group showed crypt abscess, edema, and inflammatory cell infiltration diffusing from the mucosa to the submucosa. The SASP, AND-M, and AND-H groups experienced a better therapeutic effect compared with the AND-L group. Inflammatory infiltration was observed only in the mucosa of the colon tissues but with minimal inflammatory cells in the submucosa with a mild edema.

**Figure 3:**
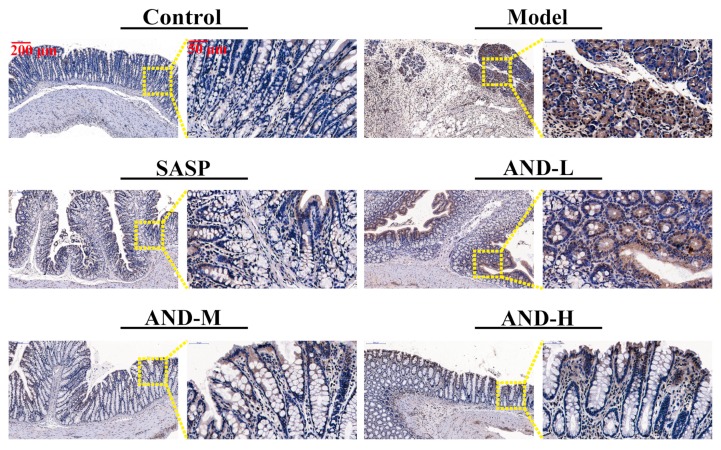
Nuclear factor kappa-B p-p65 immunohistochemistry staining in colon tissue (×200 μm and ×50 μm).

The NF-κB p-p65 antibody was used to conduct immunohistochemistry staining on paraffin-embedded colon tissues. The immunohistochemical images (×200 µm and ×50 µm) are illustrated in Figure 3. Three visual fields were randomly selected from each slice to evaluate the integrated optical density by using the Image-Pro Plus 6.0 Software, and the ratio of the integrated optical to the effective tissue area was calculated for statistical analysis (Table 2). The positive expression rate of NF-κB p-p65 in the tissues of the model group was significantly higher than that of the control group. As expected, SASP and AND treatments significantly reduced the expression of NF-κB p-p65 in the colon. The therapeutic effect of SASP was equivalent to 80 and 120 mg/kg AND.

### 2.3. MPO Content and Inflammatory Cytokine Expression

The MPO activity is an indicator of the content of tissue neutrophil cells [23]. The MPO content in the colon treated with OXZ was significantly higher compared with the control group, indicating the possible formation of oxidative damage. The MPO content in colonic tissues was significantly lower in the SASP and the AND groups after treatment for 5 days relative to the model group (Table 3). In addition, the expression levels of TNF-α, IL-4, and IL-13 were measured to further understand the extent of the inflammation development. Compared with the control group, the model group had an evident overexpression of TNF-α. Also, the TNF-α production in the AND-L, AND-H, and SASP groups was significantly lower compared with that in the model group (Table 3). In the OXZ-induced UC model, the production of IL-4 and IL-13 was upregulated significantly (Table 3). The secretion of IL-4 and IL-13 was downregulated significantly after the oral administration of SASP and 80 and 120 mg/kg AND. The expression levels of TNF-α, IL-4, and IL-13 in the colon homogenate of the AND-L group were slightly lower than those of the model group.

### 2.4. The Effect of IL-4 Receptor (IL-4R)/STAT6 Signal Pathway in OXZ-Induced UC In Vivo 

**Figure 4:**
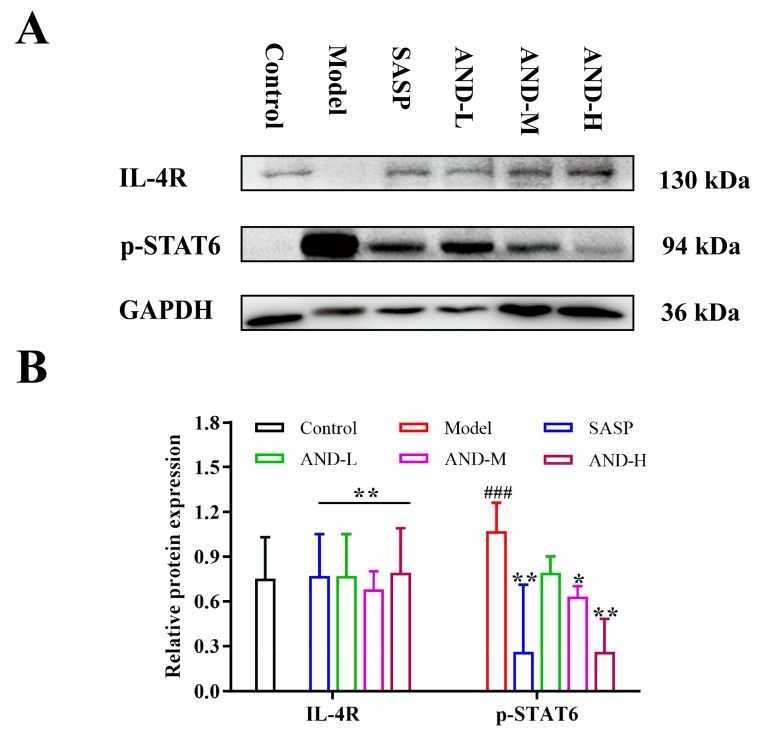
(**A**,**B**) The expression levels of IL-4R and p-STAT6. Data are represented as the mean ± SD of n = 3 (number of animals). ^###^ *p* < 0.001 compared with the control group; ** *p* < 0.01 and * *p* < 0.05 compared with the model group. (AND: andrographolide; SASP: sulfasalazine; AND-L: 40 mg/kg AND; AND-M: 80 mg/kg AND; AND-H: 120 mg/kg AND; IL-4R: interleukin-4 receptor; p-STAT6: phosphorylation of the signal transducer and activator of transcription 6; GAPDH: glyceraldehyde-3-phosphate dehydrogenase) The distributions of data obtained from the Western blot analysis were not suitable for normal analysis due to the limited number of samples.

In this study, the mechanism of AND reversing the OXZ-induced UC was explored preliminarily by using Western blot analysis. STAT6 is essential in regulating Th2-inducing cytokine production and epithelial barrier function. Also, phosphorylated STAT6 (p-STAT6) has been reported to mediate IL-4/IL-13 signaling responses [15,21,24]. Thus, the expression of STAT6 in the activated state was determined in this study (Figure 4). The p-STAT6 expression in the model group was upregulated significantly compared with that in the control group. Notably, AND and SASP inhibited the activation of STAT6. Hence, the effect of SASP was equivalent to a high dose of AND. Moreover, the inhibitory effect of AND on p-STAT6 was dose-dependent (120 mg/kg AND > 80 mg/kg AND > 40 mg/kg AND). The expression levels of IL-4R were detected in the experiment because STAT6 was activated by IL-4 and/or IL-13 binding to IL-4R. Results showed that the original IL-4R on the cell membrane was downregulated and even disappeared from some samples in the model group, which indicated the dimerization of IL-4R. SASP and AND significantly upregulated the expression of IL-4R.

## 3. Discussion

UC is almost a global disease that increases the cost of medical care and critically reduces the quality of life of patients. Unfortunately, no particular effective clinical treatment is reported because of undefined pathogenesis. Notably, the chemical components of traditional Chinese medicine, including baicalin [25], curcumin [26], berberine [27], oxymatrine [28], and astragalus polysaccharides [29], have shown significant progress in UC treatment. In addition, some studies have focused on the improvement of UC by using dietary supplements (e.g., anthocyanins, flavan-3-ols and green tea, proanthocyanidins and cocoa, isoflavones and soy, flavonols, gingerols and ginger, and hydroxycinnamic acids), nutraceutical supplements (e.g., prebiotics, probiotics, synbiotics, and fish oil), and other natural compounds in recent years [30,31,32]. For instance, gallic acid, an active component in many fruits and plants, exhibits a potential protective effect on 2,4,6-trinitrobenzene sulfonic acid-induced UC in mice [33]. Pacheco et al. investigated the anti-inflammatory bowel effect of industrial orange byproducts in dextran-sulfate-sodium-treated mice and identified pectin and phenolic compounds as the beneficial components [34]. Vitamin A has demonstrated positive clinical and endoscopic effects at a daily dose of 25,000 IU [35]. Moreover, the effect of marine natural products on UC is also under research. It has shown that caulerpin, an alkaloid from algae of the genus *Caulerpa*, can ameliorate the damage in mice colitis [36]. The meroditerpene 11-hydroxy-1’-O-methylamentadione isolated from the brown alga *Cystoseira usneoides* was effective in the protection against experimental colitis induced by dextran sulfate sodium [37]. Also, zonarol from the brown alga *Dictyopteris undulata* can protect mice against dextran-sulfate-sodium-induced UC via the inhibition of both inflammation and apoptosis [38]. It is speculated that some compounds with similar structure to zonarol may also have the effect of treating UC based on its anti-inflammatory effect, such as avarol, avarone, avarol-3’-thiosalicylate, and so on [39,40,41]. 

The American Gastroenterological Association has provided clinical practice guidelines for patients with mild to moderate UC in 2019 and identified aminosalicylates and corticosteroids as treatment for mild to moderate UC, where the right choice depends on the severity and distribution of inflammation in the colon [42]. Three types of aminosalicylates, including SASP, mesalamine, and diazo-bonded aminosalicylates, such as balsalazide or olsalazine, are mentioned in these guidelines [42]. Naganuma et al. measured the colonic mucosal concentration of 5-aminosalicylic acid in patients with UC treated with SASP and mesalamine and showed that the concentration of 5-aminosalicylic acid in the colonic mucosa of the SASP group was significantly higher than that of the mesalamine group [43]. Another study reported on the safety and efficacy of olsalazine sodium and SASP in 56 children with mild to moderate UC. Some patients belonging to the olsalazine group improved, and some showed a progression of symptoms compared with the SASP group [44]. Furthermore, SASP was selected as the positive drug on the basis of the reported studies evaluating its therapeutic effects on UC [45,46,47]. 

In the present study, the therapeutic effect of 80 mg/kg AND in improving OXZ-induced UC was better than SASP. This observation was because SASP was decomposed into 5-aminosalicylic acid, which plays a role in the treatment of UC, and sulfapyridine under the action of intestinal microbes after oral administration. However, the high-dosage administration and the long-term intake of sulfadiazine can cause severe side effects, such as headache, skin rash, blood disorders, folate deficiency, hepatotoxicity, hypospermia, and male infertility [48,49]. In addition, the experimental results suggested that the dosage of AND should be monitored to obtain the best therapeutic effect. The median lethal dose of AND in male mice is 11.46 g/kg (intraperitoneal injection), which is defined as nontoxic in mice [50]. Also, the acute toxicity test of AND has been conducted, and results have suggested that AND is safe at a maximum dose of 500 mg/kg for rats [51]. However, the animal survival rate at 120 mg/kg AND is lower than that at 80 mg/kg AND. AND may have side effects in rats with UC but has no effect in healthy rats. Therefore, the reasonable use of AND is beneficial for relieving the symptoms of UC.

OXZ was selected as the model inducer agent because of the symptomatical, morphological, and histopathological similarities between human UC and OXZ-induced UC [14]. In the rat model, pathological damage of the colon can be evaluated in accordance with literature. Majumder et al. has evaluated tissue damage in terms of inflammation, epithelium, glands, depth of lesion, and the extent of the section affected [52]. Zhang et al. has assessed histological damage by using a combined score of inflammatory cell infiltration and mucosal damage [53]. Ozsoy et al. has evaluated tissue damage only using tissue inflammation [54]. Overall, the evaluation standards of tissue damage are not uniform. Therefore, the histopathology grading system for colonic sections was modified in the present study by using the following indices: transverse section structure of the colon, degree of inflammatory cell infiltration, and tissue edema.

The mechanisms of the inflammatory immune response induced by luminal antigens in the mucosa are still unclear. The exposure of intestinal mucosa to OXZ has been reported to induce CD4^+^ T-cell-mediated delayed hypersensitivity, which results in the imbalance of immunoregulation in Th1 and Th2 subsets, and the promotion of UC progression [55]. Studies have shown that OXZ-induced colitis is an IL-4-driven model and that IL-4 production increases during disease development [56]. In addition, IL-13 can cause epithelial barrier disturbance by increasing epithelial apoptosis and upregulating the tight junction protein claudin-2 expression in the OXZ-induced colitis model [21,24,57]. Therefore, the production of IL-4 and IL-13 in the colonic homogenate of rats was measured to study changes in the OXZ-induced colitis model. Similarly, the TNF-α secreted by Th1 cells mediating the cellular immune responses detected in this paper revealed the dysfunction of immunoregulation in Th1 subsets. MPO is a functional sign and activation marker of neutrophils [23]; that is, a change in its activity represents a change in the functional state of neutrophils. The intestinal mucosa of the colon was stimulated by OXZ, resulting in neutrophil infiltration and MPO release. Under specific conditions, local antioxidants were not very resistant to the excessive oxidants produced during the catalysis of MPO, which led to oxidative stress and tissue damage. Hence, the inhibition of MPO activity and the regulation of TNF-α, IL-4, and IL-13 levels can affect the progression of UC. 

In the present study, STAT6 regulated the production of the Th2 cytokine and changed the epithelial barrier function, indicating its importance in the pathogenesis of OXZ-induced UC [21]. IL-4 and/or IL-13 can bind to their receptors and trigger a signaling cascade, leading to the phosphorylation of STAT6. Then, the p-STAT6 was dimerized and translocated to the nucleus to interact with DNA promoter elements for the regulation of gene transcription. The IL-4 receptor (IL-4R) has two types. The type I receptor is composed of the IL-4Rα and the γc chains, whereas the type II receptor consists of the IL-4Rα and the IL-13Rα1 chains. Thus, IL-4 and IL-13 are ligands for IL-4R [58]. In this experiment, the expression levels of IL-4R and p-STAT6 were detected using Western blot analysis to study the effect of AND on IL-4R–STAT6 signaling. OXZ stimulated the rat colons to overexpress IL-4 and IL-13, which resulted in the lower expression of IL-4R on the cell membrane, along with higher dimerization. As a result, a signaling cascade was triggered to upregulate the expression of p-STAT6. AND can block signal transduction by inhibiting the secretion of IL-4 and IL-13 and reduce specific binding to IL-4R. 

In summary, AND can improve OXZ-induced UC by improving the survival rate and reducing histological injury. MPO activity was attenuated after the AND treatment, reducing the oxidative damage. Moreover, the Th2 cytokines IL-4 and IL-13 and the Th1 cytokine TNF-α were involved in the inflammatory response in OXZ-induced UC. Studies on the mechanism of the anti-inflammatory effect indicated that AND can decrease inflammation effectively by regulating the balance of related inflammatory factors via the blocking of the IL-4R–STAT6 pathway in an OXZ-induced UC model. Overall, AND may be a therapeutic option for UC in the future, but some problems need to be solved. It is necessary to determine the appropriate dosage and evaluate the potential side effect of AND to achieve the desired treatment effect based on the experimental data. Moreover, the mechanism of AND reversing OXZ-induced UC should be conducted in depth to provide scientific evidence for finding a new target to cure UC. In addition, it is generally known that a convenient approach to study the pathogenesis and complexity of human UC is to induce UC in animals [14]. Various chemical agents to induce colitis models are widely used on a laboratory scale, such as dextran sodium sulphate, 2,4,6-trinitrobenzene sulfonic acid, acetic acid, carrageenan, and so on [14]. Different UC models can be used to illustrate the effect of AND on improving UC via different mechanisms. Also, the number, gender, age, and species of animals should be considered to comprehensively evaluate the effect of AND on UC in further studies. However, there are still differences between human UC and OXZ-induced UC. Accordingly, it is important to determine the therapeutic effect of AND on human UC. 

## 4. Materials and Methods

### 4.1. Chemicals and Reagents

AND (98% purity) was purchased from Guilin San Leng Biologics Co., Ltd. (Guangxi, China). OXZ (4-ethoxymethylene-2-phenyl-2-oxazolin-5-one) was provided by Sigma Aldrich Co. (St. Louis, MO, USA). Carboxymethylcellulose sodium and anhydrous ethanol were obtained from Sinopharm Chemical Reagent Co., Ltd. (Shanghai, China). Isoflurane was purchased from RWD Life Science Co., Ltd. (Shenzhen, China). Anti-IL4R Rabbit pAb (14112720) was purchased from Wanleibio (Shenyang, China). Marker, NF-κB p-p65 (ab86299), p-STAT6 (ab28829), antiglyceraldehyde-3-phosphate dehydrogenase (GAPDH), and goat antimouse IgG H&L (ab6785) antibodies were obtained from Abcam Technology (Cambridge, Cambridgeshire, UK). The bicinchonininc acid (BCA) protein quantification kit, phosphate buffered solution (PBST), radio immunoprecipitation assay (RIPA) lysis buffer, bovine serum albumin (BSA), 30% acrylic amide, 10% sodium dodecyl sulfate (SDS), N,N,N′,N′-tetramethylethylenediamine (TEMED), ammonium persulfate (APS), and sample loading buffer (5X) were purchased from YEASEN Biotechnology Co., Ltd. (Shanghai, China). Protease and phospholipase inhibitors were obtained from Roche Applied Science (Foster City, CA, USA). Poly (vinylidene fluoride) (PVDF) membrane and the Immobilon^TM^ Western chemiluminescent horseradish peroxidase (HRP) substrate were purchased from Millipore (Billerica, MA, USA). Methanol (HPLC grade) was purchased from Fisher Scientific Co. (Santa Clara, CA, USA). Deionized water was purified using the Milli-Q Academic System (Millipore Corp., Billerica, MA, USA).

### 4.2. Animals

A total of 66 healthy male Sprague Dawley rats (body weight: 200–250 g) were obtained from the Drug Safety Evaluation and Research Center of the Shanghai University of Traditional Chinese Medicine (Shanghai, China). All rats were raised under a specific pathogen-free environment and provided free access to water and rodent chow for one week before the experiment. Animals were kept in a climate-controlled environment set to 25 ± 2 °C and on a 12-h light/dark cycle. Animal studies were conducted in accordance with the regulations for animal experimentation issued by the State Committee of Science and Technology of the People’s Republic of China on 14 November 1988 and approved by the Animal Ethics Committee of the Shanghai University of Traditional Chinese Medicine (No. PZSHUTCM18122111, Approval date: 21 December 2018).

### 4.3. Induction and Assessment of OXZ-Induced Colitis

**Figure 5:**
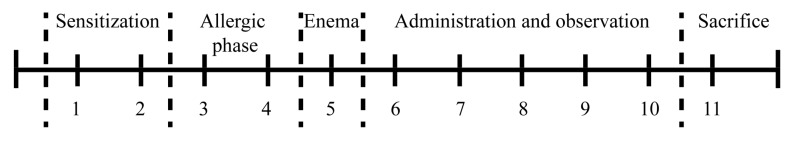
The time schedule of the experiment.

The method used to induce colitis using OXZ has been reported previously [56,59]. The process was divided into three phases, namely sensitization, allergic phase, and enema (Figure 5). For sensitization, rats were lightly anesthetized with isoflurane, and 300 μL OXZ (30 mg/mL, anhydrous ethanol) was applied on the shaved back within a field of approximately 2 cm × 2 cm. Meanwhile, anhydrous ethanol without OXZ was dripped onto the exposed skin in the control group. On the fifth day, rats were subjected to intraperitoneal anesthesia with 10% chloral hydrate (300 mg/kg). Then, a catheter (diameter: 2 mm; length: 12 cm; silica gel flexible pipe) was inserted into the anus at a depth of about 8 cm, and 300 μL OXZ (30 mg/mL, 50% ethanol/water mixture solution) was injected using a 1 mL syringe. The rats were held in a vertical position for 60 s after the injection to avoid leakage of OXZ from the anus.

Animals treated with OXZ were randomly divided into five groups (n = 11 per group) on the next day in accordance with the fecal traits: sulfasalazine + OXZ group (labeled as SASP, 500 mg/kg, positive control group), OXZ group (labeled as model), AND + OXZ groups (40, 80, and 120 mg/kg, labeled as AND-L, AND-M, and AND-H, respectively). AND and SASP were suspended in a 0.5% methylcellulose solution. The rats belonging to the control and the model groups were administered with 2 mL 0.5% methylcellulose via oral gavage once per day. Body weight loss, stool consistency, and the bloody stool of animals were recorded daily to assess the development of experimental colitis using the DAI scoring system [59,60]. The scoring rules were as follows: body weight loss (0: normal; 1: 1–5%; 2: 6–10%; 3: 11–15%; 4: >15%), stool consistency (0: normal; 2: loose stools; 4: diarrhea), and bloody stool (0: negative; 2: positive; 4: gross bleeding). The formula for calculation of DAI is as follows: DAI = (body weight loss score + stool consistency score + bloody stool score)/3.

The rats were sacrificed after six days of OXZ administration, and the spleen coefficient and colon length were measured. The colon was opened longitudinally and flushed with ice-cold normal saline. The intestinal segments were collected at a distance of 6 cm from anus and fixed in 4% paraformaldehyde for 24 h. The fixed samples were then embedded in paraffin wax, stained with H&E for histopathological analysis in accordance with previously published literature [52,53,54] with some modifications. The first step was to observe whether the transverse structure of the colon was intact (intact: 0; mucosa damage: 1; submucosa damage: 2; muscle damage: 3). Second, the inflammatory infiltration in the colon was evaluated (no or a few inflammatory cells was designated as 0; many inflammatory cells in the mucosa layer was designated as 1; confluence of inflammatory cells extending into the submucosa was designated as 2; transmural extension of the inflammatory cell infiltration was designated as 3). Third, the degree of edema of the colon tissue was evaluated (normal: 0; mild edema: 1; moderate edema: 2; severe edema: 3). Moreover, the NF-κB p-p65 antibody was used to stain the paraffin-embedded colon tissue for immunohistochemistry analysis, and results were evaluated using the Image-Pro plus 6.0 (Media Cybernetics, Bethesda, MD, USA). The clean colon was cut into two parts longitudinally and stored at −80 °C. One part was used to detect the expression levels of related inflammatory factors, and the other part was used for Western blot analysis.

### 4.4. MPO, TNF-α, IL-4, and IL-13 Assay

The colon tissues were homogenized using a phosphate buffer solution (pH 7.4) and centrifuged at 10,000 rpm for 10 min at 4 °C. The supernatant was collected to measure the expression levels of MPO, TNF-α, IL-4, and IL-13 by using enzyme linked immunosorbent assay (ELISA) kits in accordance with the manufacturer’s instructions (Nanjing SenBeiJia Biotechnology Co. LTD., Nanjing, China). Particularly, the expression levels of the above inflammation-related factors in the colon homogenate were normalized using the protein concentration of each sample.

### 4.5. Western Blot Analysis

The colon tissues were homogenized using the RIPA lysis buffer containing protease and phosphatase inhibitors. The protein lysate was harvested via centrifugation at 10,000 rpm for 10 min at 4 °C, and the total protein content was determined using the BCA protein assay kit. The proteins were then mixed with a loading buffer and heated at 95 °C for 5 min. Approximately 20 µg protein was separated using 10% SDS–PAGE and transferred to PVDF membranes. After blocking with 5% fat-free milk in PBST at room temperature, the membranes were incubated with IL-4R (1:500), p-STAT6 (1:750), and GAPDH (1:2000) antibodies overnight at 4 °C. Subsequently, the membranes were incubated with HRP-conjugated antirabbit (1:5000) secondary antibodies for 2 h at room temperature. The protein bands were visualized using the electrochemiluminescence (ECL) prime kit and analyzed using the GS-700 imaging densitometer (Bio-Rad Laboratories, Hercules, CA, USA). The expressions of the target proteins were quantified following normalization to the expression of GAPDH.

### 4.6. Data Analysis

The IBM SPSS statistics 21.0 (International Business Machines Corporation, Armonk, NY, USA) and the Prism 5.0v software (GraphPad Software Inc., San Diego, CA, USA) were used to analyze the data. The Kolmogorov–Smirnov tests were used to determine data normality (small sample). One-way analysis of variance (ANOVA) and a two-independent-sample nonparametric test (Mann–Whitney test) were applied to compare the differences among groups. The values of *p* < 0.05, 0.01, and 0.001 were considered statistically significant.

## Figures and Tables

**Table 1:** Disease activity index (DAI) of experimental rats.

	Control	Model	SASP	AND-L	AND-M	AND-H
1	0.00 (0.67, 0.00)	2.00 (2.67, 0.67) ^###^	2.00 (2.67, 1.33)	2.00 (2.67, 0.67)	2.00 (2.67, 0.67)	1.33 (2.67, 0.67)
2	0.00 (0.67, 0.00)	1.67 (2.67, 0.67) ^###^	1.33 (2.00, 0.00) *	1.33 (2.00, 0.00)	1.00 (2.00, 0.00) **	1.00 (2.33, 0.67) *
3	0.33 (0.67, 0.00)	1.50 (2.33, 0.67) ^###^	0.67 (2.33, 0.00) *	1.33 (1.67, 0.00)	0.67 (1.67, 0.00) **	1.00 (1.67, 0.00) *
4	0.00 (0.67, 0.00)	1.33 (1.67, 0.67) ^###^	0.67 (2.33, 0.00) *	1.17 (1.67, 0.00)	0.67 (1.00, 0.00) *	0.67 (1.67, 0.00) *
5	0.00 (0.67, 0.00)	0.67 (1.33, 0.67) ^###^	0.67 (0.67, 0.00) *	0.67 (2.33, 0.00)	0.67 (1.00, 0.00) *	0.00 (1.00, 0.00) *

Notes: The data distribution of DAI did not accord with the normal distribution, so the data are presented as median (maximum value, minimum value). ^###^ *p* < 0.001 vs. control group; * *p* < 0.05 vs. model group; ** *p* < 0.01 vs. model group. (DAI: Colonic disease activity index; AND: Andrographolide; SASP: Sulfasalazine; AND-L: 40 mg/kg AND; AND-M: 80 mg/kg AND; AND-H: 120 mg/kg AND.) The calculation method of DAI is shown in the “Materials and Methods” section.

**Table 2:** Density of positive cells.

Groups	Control	Model	SASP	AND-L	AND-M	AND-H
Density	3.03 ± 0.96	46.35 ± 11.54 ^###^	7.71 ± 3.41 ***	33.44 ± 2.35 *	12.51 ± 1.75 ***	14.47 ± 3.88 ***

Notes: Data were presented as means ± SD, n = 3 (number of animals). ^###^ *p* < 0.001 vs. the control group; *** *p* < 0.001, * *p* < 0.05 vs. the model group. (AND: andrographolide; SASP: sulfasalazine; AND-L: 40 mg/kg AND; AND-M: 80 mg/kg AND; AND-H: 120 mg/kg AND.) The distributions of data obtained from immunohistochemical were not suitable for normal analysis due to the limited number of samples.

**Table 3:** MPO, TNF-α, IL-4, and IL-13 content.

Groups	MPO	TNF-α	IL-4	IL-13
Control	43.40 ± 6.47	80.29 ± 15.46	31.00 ± 5.51	10.79 ± 1.38
Model	54.14 ± 6.92 ^###^	105.49 ± 12.49 ^###^	37.07 ± 4.72 ^##^	12.80 ± 1.46 ^##^
SASP	40.09 ± 4.81 ***	86.95 ± 10.98 **	32.50 ± 2.53 *	10.61 ± 1.39 **
AND-L	47.32 ± 3.83 *	99.83 ± 11.09	33.70 ± 3.28	12.26 ± 1.29
AND-M	44.49 ± 6.23 **	91.29 ± 9.95 *	30.59 ± 4.39 **	10.80 ± 1.49 **
AND-H	44.70 ± 6.13 **	91.11 ± 6.63 *	30.54 ± 2.69 **	10.13 ± 0.91 ***

Notes: Data obtained from experiments conformed to the normal distribution, so results are shown as means ± SD, n = 7–11 (control and AND-M groups: n = 11; model group: n = 7; AND-L, AND-H, and SASP group: n = 9). Compared with the control group, ^###^ *p* < 0.001, ^##^ *p* < 0.01; relative to the model group, *** *p* < 0.001, ** *p* < 0.01, * *p* < 0.05. (AND: andrographolide; SASP: sulfasalazine; AND-L: 40 mg/kg AND; AND-M: 80 mg/kg AND; AND-H: 120 mg/kg AND; MPO: myeloperoxidase; interleukin-4: IL-4; interleukin-13: IL-13; tumor necrosis factor-α: TNF-α).
